# Supplementary material for: A blank check or a global public good? A qualitative study of how ethics review committee members in Colombia weigh the risks and benefits of broad consent for data and sample sharing during a pandemic
Source: PLOS Glob Public Health. 2022 Jun 6;2(6):e0000364. doi: 10.1371/journal.pgph.0000364 (PMC10022129; doi:10.1371/journal.pgph.0000364)
Supplement: S1 Table — (PDF) [file pgph.0000364.s001.pdf]

S1 Table. Themes related to broad consent for future use and representative quotes from ethics committee members

Theme	Representative Quote - Spanish	Representative Quote - English
**(+) Benefits of broad consent for future use**		
(CEI-016, female, President, over 45 yrs. old)	"Hay un beneficio para.. la cura de futuras enfermedades, si es que estamos hablando de enfermedad o para las futuras generaciones también. Y por otro lado, está el beneficio que está en entrenar nuevos investigadores en esas temáticas, indirectamente también beneficiaría a las comunidades porque tendríamos más investigadores debidamente entrenados."	"There is a benefit for ... the cure of future diseases, if we are talking about disease or for future generations as well. And on the other hand, there is the benefit of training new researchers in these topics, indirectly it would also benefit the communities because we would have more researchers properly trained. "
(CEI-026,-female, Chairperson, over 45 yrs.old)	"Yo lo veo muy pertinente precisamente porque tener un consentimiento ampliado permite que nosotros tengamos un buen aprovechamiento...pues..de los recursos. Sobre todo en este país donde es tan difícil conseguir recursos para investigación, y que conseguir las muestras lleva un buen porcentaje del dinero de la investigación."	"I see it as very pertinent precisely because having broad consent allows us to have a good use...well...of resources. Especially in this country where it is so difficult to get resources for research, and that obtaining the samples takes a good percentage of the research money."
**(+) Utility for pandemic response**		
(CEI 014, female, Chairperson, over 45 yrs. old)	"Específicamente, por ejemplo, en casos de infecciones, de pandemias como ahora, que estamos seguros que no se hubieran podido desarrollar las vacunas tan rápidamente, si no hubiesen unos biobancos y repositorios de datos disponibles para poder hacerlo, entonces la ventaja es que hay información, hay suficiente para investigar y para trabajar pues, en lo que se requiera, están disponibles, ese es el mayor beneficio."	"In cases of infections, pandemics like now, we are sure that vaccines could not have been developed so quickly, if there were not biobanks and data repositories available to do so, then the advantage is that there is information, there is enough to investigate and work because, in what is required, it is available, that is the greatest benefit."
**(+) Importance of big data**		
(CEI-017, male, President, over 45 yrs. old)	"Ya no podemos pensar que un grupo de investigación aislado hace un descubrimiento y con eso lo valida todo, estamos en el mundo del big data. La bioestadística nos está mostrando que muchas veces somos más efectivos cuando estamos unidos adecuadamente y esto va a implicar una homologación."	"We can no longer think that an isolated research group makes a discovery and with that validates everything...we are really in the world of big data. Biostatistics is showing us that many times we are more effective when we are properly united and this will imply a homologation."
(CEI-009, male, Chairperson, over 45 yrs. old)	"Como beneficiario la sociedad, la humanidad , pues es posible hacer ese uso común de esa información, de esa muestra, pero...de ahí a que eso sea, tener un sentido altruista del propósito... del uso de esas muestras."	"It is possible to make use of that information, of that sample, for the benefit of society, of humanity. To make that possible one has to have an altruistic sense of the purpose for the use of those samples."
**(+) Benefits of genomic data**		

Theme	Representative Quote - Spanish	Representative Quote - English
**sharing for specific populations**		
(CEI-003, male, President, 35-45 yrs. old)	"Tiene muchos beneficios, identificación de enfermedades ligadas a genes asociados al genotipo de esa comunidad, efectos de medicamentos, efectos de una vacuna o efectos de una enfermedad infecciosa o no infecciosa sobre ese tipo de genética o de genoma."	"It has many benefits, identification of diseases linked to genes associated with the genotype of that community, effects of drugs, effects of a vaccine or effects of an infectious or non-infectious disease on that type of genetics or genome."
**(-) fear of coercive consent**		
(CEI-023, female, community representative, over 45 yrs. old)	"Cuando usted firma, que era lo que le explicaba...cuando firma ese consentimiento, al paciente no le haya quedado muy claro hasta dónde ¿no? el alcance, y puede haber un riesgo de que él no deseara que esa investigación o el resultado o sus datos sean utilizados más allá de lo que él entendió inicialmente que se podía utilizar ¿no?"	"When you signed, which was what I explained to you (...) when you signed that consent, it was not very clear to the patient how far, right? the scope, and there may be a risk that he would not want that research or the result or his data to be used beyond what he initially understood could be used, right?"
(CEI-009, male, Chairperson, over 45 yrs. old)	"Entonces... qué es un individuo competente, pues ahí nos dice, ah pues es un individuo que física y moral, moralmente pues está en sus cabales. Pero yo le agregaría algo más, lo cultural, pues es muy fácil y uno lo ve... construir un consentimiento en donde usted no entienda nada, y en algunos casos casi que ni sepa leer o escribir y le digan... si está de acuerdo, fírmeme aquí, y el problema que viene ahí es el de la libre decisión, si? "	"So...what is a competent individual?...an individual who is physically and morally...in his right mind. But I would add something else, the cultural, because it is very easy and you see it...to build a consent where you do not understand anything, and in some cases the person almost doesn't know how to read or write and they ask him, if he is in agreement, to sign there and...the problem that comes there is that of free decision."
**Need for clarity in informed consent language**		
(CEI-004, male, Chairperson, 35-45 years old)	"Un riesgo es a veces el desconocimiento de los procesos de investigación, que los llevan a un bajo entendimiento de los consentimientos informados. No es nuevo que cuando se realizan investigaciones hay que hacer un esfuerzo grande para que quede claro el consentimiento informado a los participantes. Y, a veces, ese contexto de los consentimientos amplios, pues terminan derivando en la buena fe o en la esperanza que tiene el voluntario en la buena fe de los investigadores, ¿sí?"	"A risk is, sometimes the lack of knowledge of the investigation processes, which leads them to a low level of understanding of the informed consents. It is not something new that when research is conducted, you have to really make an effort to ensure that the informed consent is clear to the participants. And sometimes, in this context of broad consent, it ends up depending on good faith or on the participant's hope for the investigator's good faith. Correct?"
(CEI-010, male, Chairperson, over 45 yrs. old)	"una cosa que creo es el caballo de batalla dentro del comité de ética, es que los consentimientos sean no suficientemente claros, para que una persona con el nivel de escolaridad que se espera que sea el paciente, lo pueda leer y saber qué está pasando...los derechos de las personas deben ser respetados, lo cual suena como circular a más no poder, y esa sociedad tiene que respetar la individualidad y para respetarla, los consentimientos deben estar bien formulados."	"The [Trojan] horse within the ethics committee is that the consents are not sufficiently clear, so that a person with the level of education that the patient is expected to have can read the consent and know what is happening... People's rights must be respected, society must respect the individual and...to respect them, consents must be well formulated".

Theme	Representative Quote - Spanish	Representative Quote - English
(CEI-009, male, Chairperson, over 45 yrs. old)	“Entonces... qué es un individuo competente, pues ahí nos dice, ah pues es un individuo que física y moral, moralmente pues está en sus cabales. Pero yo le agregaría algo más, lo cultural, pues es muy fácil y uno lo ve... construir un consentimiento en donde usted no entienda nada, y en algunos casos casi que ni sepa leer o escribir y le digan... si está de acuerdo, fírmeme aquí, y el problema que viene ahí es el de la libre decisión, si?”	“So...what is a competent individual?...an individual who is physically and morally...in his right mind. But I would add something else, the cultural, because it is very easy and you see it...to build a consent where you do not understand anything, and in some cases the person almost doesn’t know how to read or write and they ask him, if he is in agreement, to sign there and...the problem that comes there is that of free decision."
**Need for benefit sharing**		
(CEI-001, female, Vice President, over 45 yrs. old)	“Si aquí vienen los estudios para la vacuna covid a menos 70 grados, que hay que conservar a menos 70 grados, pues a mí me da mucha pena, podrá ser la vacuna para el COVID, pero ¿aquí en donde tenemos congelación a menos 70?”	“Here come the researchers for the COVID vaccine...which must be kept at minus 70 degrees...Here, where do we have freezing at minus 70?”
(CEI-017, male, President, over 45 yrs. old)	“es porque queremos también evitar este uso de muestras en Latinoamérica de hacer investigación extractiva. Es decir, Latinoamérica como dispensador de muestras...solamente un recolector.”	“We also want to avoid this use of samples in Latin America to do extractive research. That is to say, Latin America as a sample dispenser only...only as a collector.”
(CEI-013, male, Chairperson- over 45 yrs. old)	“Y si estas muestras provienen de comunidades vulnerables, digamos si proviene de gente en condiciones de pobreza, gente en condiciones de vulnerabilidad de tipo étnico o racial... Nosotros ahí tenemos una mirada un poco...crítica y cuestionadora, frente a... ¿cuál es el rol frente a ese tipo de solicitudes? ¿Por qué quieren ampliar el consentimiento? ¿Por qué no trabajan con lo que se tiene o hasta donde solicitaron el consentimiento?”	"If these samples come from vulnerable communities, let's say if they come from people in conditions of poverty, people from vulnerable ethnic or racial groups, in that case we take a critical view, we question...What is the role of this type of request? Why do they want to extend consent? Why don't they work with what they have...or to what extent did they request consent?"
(CEI-016, female, President, over 45 yrs. old)	“Entonces las tecnologías que existen en el país o en la institución no están disponibles, entonces tiene que trabajar con otras instituciones.”	“Technologies that exist in that country or in that institution are not available, so one has to work with other institutions”
(CEI-004,male,Chairperson, 35-45 years old)	“¿y si logran identificar alguna alteración clínica identificada de esas muestras? ah bueno…permitan el consentimiento amplio pero...pero si en las muestras se encuentra alguna condición inherente, pregunten al paciente si quiere conocer esa información inherente de sus muestras, o sea, es una desvinculación total de relación de las muestras versus la investigación, que... desde por lo menos mi punto de vista, es que el paciente termina siendo como un insumo, un proveedor de información más no hay una interacción ni un beneficio que pueda derivar para él”. Yo creo que va muy bien marcado, cómo a ser sinceros con los voluntarios en hacia dónde va el enfoque, y que también el voluntario tenga capacidad de definir si él quiere ese beneficio o no quiero ese beneficio.”	"What if they manage to identify a clinical alteration in those samples?...well...allow broad consent, but...if any inherent condition is found in the samples, ask the patient if they want to know that inherent information from their samples…It is this total dissociation of the relationship of samples versus research, which...is that the patient ends up being an input, another provider of information, there is no interaction or benefit...that can derive for him.”
**Concerns about appropriate governance**		

Theme	Representative Quote - Spanish	Representative Quote - English
(CEI-004, male, Chairperson, 35-45 years old)	“Algo que no aprobaría, es dejar el consentimiento informado amplio como un cheque en blanco, una cosa que quede como...libre.”	"Something that I would not approve of is to leave the broad informed consent like a blank check, something that remains like...open."
(CEI-010, male, Chairperson, over 45 yrs. old)	“Los biorepositorios son utilizados como una fuente, como una puerta de atrás para hacer ciertas cosas."	"Biorepositories are used as a source, as a backdoor to do certain things."
(CEI-004, male, Chairperson, 35-45 years old)	“ quién salvaguarda la información … Quién es el encargado de definir que esa muestra sale o no sale para investigación, y el doliente directo, de pues, administrar y salvaguardar su información. Otra es un área definida de... un límite, por decirlo así a ese consentimiento ampliado, ¿sí? Por lo menos un área temática, o por lo menos una enfermedad definida, o por lo menos un contexto definido, no que quede algo como abierto.”	“Who safeguards the information?...Who is in charge of defining whether this sample leaves or does not leave for investigation, and... the person directly affected, then, to administer and safeguard their information? Another [point] is a defined area... a boundary, so to speak, to that expanded consent, yes? At least a subject area, or at least a defined disease, or at least a defined context, not something that remains open.”
(CEI-017, male, President, over 45 yrs. Old)	“Justamente la figura de un biobanco fortalecido, bien regulado, con buenas voluntades por supuesto también (…) a lo mejor permite, como hacer más importante el objetivo de la cooperación científica.”	“Precisely the figure of a strengthened, well-regulated biobank, with good will…it makes the objective of scientific cooperation more important.”
(CEI 014, female, Chairperson, over 45 yrs. old)	“Creo que no queda plasmado suficientemente como yo desearía que estuviera. Cumple, digamos, que el protocolo cumple con el estándar, cumple con una norma que dice, si, es... identifican el sitio donde estarán guardadas las muestras, los biobancos, los repositorios, donde están, en qué ciudad. Pero no es muy claro... Yo pensaría que debe hacerse mayor énfasis en eso, en eso... en la gobernanza.”	“I think it is not written down as much as I wish it were...Let's say, that the protocol complies with the standard, it complies with a norm that says, yes,...it is that they identify the place where the samples will be stored, the biobanks, the repositories, where they are, in what city. But it is not very clear... I would think that...greater emphasis should be placed on that, on that...the governance”
**Consideration of sharing with academic or public health versus industry partners**		
(CEI-008, male, community representative, over 45 yrs. old)	“En los estudios de investigación a nivel institucional, a nivel educativo, a nivel de universidades, es más sano.. A nivel de la industria porque se mueven intereses muy grandes, políticos, de...de grandes financieras...entonces ahí se presta para cualquier desviación, para cualquier abuso”	“In research studies at the educational level, at the university level, it is healthier. At the industry level, because political and financial interests are involved, it lends itself to any deviation, to any abuse."
(CEI-005, male, Chairperson, 35-45 years old)	“pero el del investigador (el interés) es poder producir y poder generar su trabajo y su reputación. Otra cosa es mirar las instituciones involucradas, o sea, cuáles son las partes involucradas y qué quieren hacer con el resultado, o sea, para dónde van esos resultados, a quienes están dirigidos.”	“but the researcher's [interest] is to be able to produce and build their work and reputation. Another thing is to look at the institutions involved, that is, who are the parties involved and what do they want to do with the results, that is, where do these results go, to whom they are addressed.”

Theme	Representative Quote - Spanish	Representative Quote - English
(CEI-001, female, Vice President, over 45 yrs. old)	“Entonces, independientemente de que sea un patrocinador que pueda tener interés económico o una institución de salud pública, sobre todo la industria, pueden hacer uso indebido. ¿Para qué la van a querer (la muestra biológica)? van a obtener un producto, y quienes están poniendo sus muestras, nunca van a tener ningún beneficio, ¿sí?”	“So regardless of whether it is a sponsor that may have an economic interest or a public health institution, especially industry, can misuse it. Why would they want it (the biological sample)? they're going to get a product, and whoever's putting up their samples, they're never going to make any profit, okay?”
**How COVID-19 has affected the ethics review process**		
(CEI-001, female, Vice President, over 45 yrs. old)	“Creo que en la burocratización de la investigación y de los comités de ética nos volvimos paquidérmicos, todos nos volvimos paquidérmicos, entonces es carta va carta viene, cite reunión, y nos demoramos unos tiempos que… no tendrían que ser necesarios y la pandemia nos ha demostrado que, efectivamente, es así, que podemos ser más eficientes, y que podemos evaluar en 3 días y que podemos, [este], tener aprobaciones en ese periodo de tiempo.”	“I think that in the bureaucratization of the investigation and of the ethics committees we became pachydermic...we delayed for a time that...should not be necessary. The pandemic has shown that...we can be more efficient, and that we can evaluate in 3 days...we can...have approvals in that period of time."
**Data ownership**		
(CEI-001, female, Vice President, over 45 yrs. old)	“La muestra que se me toma hoy a mi con una identificación, etc., etc., pues me pertenece a mí, esa muestra va a permitir generar una gran cantidad de información y de datos, que sumado con otras va a producir investigación y eso pues…le pertenece o nos pertenece. Lo que se genere de allí, digámoslo así, les pertenece a todos ¿sí? A todos los que participaron, a los que participaron en la generación del proyecto, y en general a la humanidad porque es la generación del conocimiento que debería, que debe ser universal y global y accesible para todos.”	"The sample that is taken from me today with an identification, etc., etc., well, it belongs to me, that sample will allow for the generation of a large amount of information and data, which, together with other samples will produce research…and that, well...it belongs to him or it belongs to us. Whatever is generated from that, let's say it like this, it belongs to everyone. Yes? To all those who participated, to those who participated in the generation of the project, and in general to humanity because it is the generation of knowledge that should, which must be universal and global and accessible to all."
(CEI-001, female, Vice President, over 45 yrs. old)	“Y creo que esta pandemia nos lo ha puesto en la cara, no es local, ni regional, ni mía, ni el conocimiento es mío, sino que justamente es de todos, todos tenemos que construirlo, todos tenemos que poner, a todos nos va a costar, ¿sí? Creo que, de las enseñanzas de la pandemia, justamente en este campo es esa.”	"And I believe that this pandemic has put it in our faces, it is not local, not regional, not mine, nor is the knowledge mine, but rather belongs to everyone, we all have to contribute, we all have to put it, it costs us all. Yes? I think that, in [the research] field, that is what we have learned from the pandemic.”
**Community engagement**		
(CEI-001, female, Vice President, over 45 yrs. old)	“tenemos que generar espacios para educar a la comunidad y a las personas en investigación. Y esta pandemia nos ha mostrado eso, ¿sí? esta pandemia ha expuesto la necesidad de hacer investigación, ha mostrado la… digamos…en nuestro medio el poco conocimiento, y la necesidad de educar y también, el interés que realmente las personas pueden, y realmente las comunidades pueden tener en conocer acerca de…lo que es la investigación, la ética en investigación, los procesos que están en todo esto, los procesos de	“We have to create spaces to educate the community and people in research. This pandemic has exposed the need to do research, has shown...in our environment the little knowledge, and the need to educate and also, the interest that people...and...communities can have in knowing about…what research is, research ethics, the processes that are in all this, the informed consent processes, etc. So I believe that people should absolutely be involved in an educational process that allows them to participate in the decision making

Theme	Representative Quote - Spanish	Representative Quote - English
	consentimiento informado, etc. Entonces creo que definitivamente las personas deben ser involucradas en un proceso educativo que les permita ser partícipes de toma de decisiones en lo que respecta a… a la generación de conocimiento justamente a través de lo que ellos son, viven, de los problemas que se tienen, de lo que…de los recursos que ellos aportan, como por ejemplo sus muestras.”	process related to the generation of knowledge related to how they are, their lives, the problems that they have, the resources they provide, like their biological samples."
(CEI-017, male, President, over 45 yrs. old)	“Nosotros hacíamos pequeñas cosas, muy fragmentadas como una operación hormiga de investigación, y después por acumulación quedaba un corpus de conocimiento. No, ahora ha ido cambiando esto porque entendemos que la investigación seria, estructurada, requiere mucho tiempo, hacer una secuenciación, estar al lado de esas comunidades, por ejemplo, trabajar con las comunidades. Creo que eso ha madurado el pensamiento de nuestros investigadores para tener una proyección más grande.”	“We used to do little [projects], very fragmented like a research ant operation, and those projects accumulated and we were left with a body of knowledge. Now, the research enterprise has changed. We know that serious research will be structured, require a lot of time, will go in sequence, will be accompanied by these communities, with work with these communities. I think our researchers' thinking has matured to include a larger picture.”
(CEI-012, female, Chairperson, 35-45 yrs. old)	“Son los investigadores los que proponen ese consentimiento informado ampliado, nunca hemos visto la participación de la comunidad o de la sociedad civil, por ejemplo, que esté involucrada en los proyectos. Casi nunca los proyectos llegan a la mesa de discusión planteados desde la comunidad o con participación de actores diferentes a los investigadores. Eso también debería cambiar, ¿no? porque desde el planteamiento de los problemas de esos proyectos, se le debería dar mayor participación a la gente, a la gente del común, no solo a los investigadores.”	“The researchers are the ones who propose this broad informed consent. We have never seen the participation of the community or civil society, for example, their involvement in these projects. Research projects that are presented for discussion [by the ERC] are almost never suggested by the community or by anyone other than the investigators themselves. That should change too, right? From the proposal of the research objectives, people should be given a bigger role, common people, not just the investigators themselves.”
**Role of community members on the ERC**		
(CEI-007, female, community representative, over 45 yrs.old)	“Por ser representante de la comunidad, debo leer un consentimiento informado...como no pertenezco al gremio de la salud, debo entenderlo, en sus palabras, en su contexto, en todo... entonces, yo no reviso todo el protocolo, yo solamente leo los consentimientos.”	"As a representative of the community, I must read an informed consent...as I do not belong to the health field, I must understand it, the words, the context, everything...so, I do not review the entire protocol, I only read the consents”
(CEI-019, male, Chairperson, 35-45 yrs. old)	“Pues es que casi siempre lo que me preguntan es si entendí el consentimiento.”	“Almost always they ask me if I understood the consent.”
**Lack of national guidance on broad consent for future use**		
(CEI-020, male, President, 35-45 years old)	“Digamos aquí como no hay una ley de biobanco o una norma específica. Digamos que hay unos referentes indirectos que no son referentes en investigación pero ayudan.”	“ Let's say here how there is no biobank law or a specific norm. Let's say that there are some indirect referents that are not referents in research but help/”

Theme	Representative Quote - Spanish	Representative Quote - English
(CEI-020, male, President, 35-45 years old)	“No tenemos de dónde agarrarnos normativamente, no hay,digamos que es clave discutirlo en algún momento.”	"We don't have anything to hold on to normatively, there is nothing. Let's say it is important to discuss this at some point."
**Individual beliefs versus group process**		
(CEI-017, male, President, over 45 yrs. old)	“Yo creo que el problema de muchos comités es que borran la voz del otro, y por eso hay ofensa. Hay ofensa porque el investigador se siente ninguneado, utilizando esta expresión coloquial, pues...o degradado.”	I believe that the problem with many committees is that they erase the voice of the other, and that is why there is offense. There is offense because the researcher feels ignored, using this colloquial expression, well ... or degraded.”
**Need for additional training**		
(CEI-012, Female, Chairperson, 35-45 yrs old)	“no tengo mucha claridad a que se refiere el consentimiento informado amplio, si es como un consentimiento general donde no se especifica con mucha profundidad las características de proyecto y como los deberes y derechos a los cuales tiene el individuo o si está referido a pedirle el individuo el uso posterior de...para otros proyectos de investigación.”	“I am not very clear about what broad informed consent refers to, if it is a general consent wherein the characteristics of the project are not specified in great detail and like the individual’s duties and rights or if it refers to asking the individual for the subsequent use of [their data] for other research projects.”
(CEI-006, female, community representative, 35-45 years old)	“Pues yo lo entendería (definición de CIA) como aquel documento en el que el participante realmente se va a enterar de cómo es el estudio, que tiene que hacer esta persona, cual son los beneficios que puede o no obtener de participar en este estudio... y que es lo que le van a solicitar, que entregue o haga esa persona que desea participar en este estudio.”	“Well, I would understand it (ICF definition) as that document in which the participant is really going to find out what the study is like, what this person has to do, what are the benefits that they can or cannot obtain from participating in this study (...) and what are they going to request, that the person who wishes to participate in this study deliver or do.”
(CEI-003, male, President, 35-45 yrs. old)	“Quiero preguntar si el concepto de consentimiento amplio que está manejando en esta encuesta es un consentimiento que no...que no vence. Nosotros siempre recibimos de la industria solicitudes y el consentimiento por lo general vence, es decir , por los próximos cinco años vamos a compartir datos, pero dicen que a los 5 años. Teóricamente ese es un consentimiento amplio pero limitado, limitado en el sentido de temporal, porque los consentimientos informados amplios ilimitados creo que no existen.”	“I want to ask if the concept of broad consent that you are using in this survey is a consent that does not expire ...We always receive requests from the industry and consent usually expires that is..., for the next five years we are going to share data, but they say that after 5 years. Theoretically, this is a broad but limited consent, limited in the sense of temporary, because I believe that unlimited broad informed consents do not exist.”

Recordings and notes from in depth interviews were transcribed verbatim by data collectors, deidentified, and then analyzed in Spanish. (+) denotes a statement about the benefits of broad consent for future use. (–) denotes a statement of concern about broad consent for future use. No demarcation is used for neutral statements.
