# Supplementary material for: A blank check or a global public good? A qualitative study of how ethics review committee members in Colombia weigh the risks and benefits of broad consent for data and sample sharing during a pandemic
Source: PLOS Glob Public Health. 2022 Jun 6;2(6):e0000364. doi: 10.1371/journal.pgph.0000364 (PMC10022129; doi:10.1371/journal.pgph.0000364)
Supplement: S2 Text — (PDF) [file pgph.0000364.s003.pdf]

S1 File. Semi-structured in-depth interview guide

Previous training / experience

1. What were your motivations for participating in this ethics review committee?

Approach to assessing broad informed consent for future use of biological samples and data

2. From your point of view, who do you consider to be the stakeholders (or actors) when you are evaluating research protocols that include a statement about the sharing of unidentified data for purposes other than those described in the original research study, in other words, broad informed consent for future use of the data or samples?

Example: If you consider group 1 of stakeholders to be: funders and researchers and group 2: research participants and the communities from which they come.

Probe:

- How are the interests of GROUP 1 and GROUP 2 different?

3. Describe a time when the interests of the different actors involved in a research project have been in conflict.

Probe:

- How did you assess / or weigh the concerns and interests of different parties / groups?
- What helped you take a position or a decision in the face of those concerns or conflicts?

4. In your opinion, what are the greatest risks for the participants and their communities, derived from or associated with the fact that the researchers share / use their anonymized data, in other investigations different from the original investigation.[1]

Probe:

Do you think these risks are different according to:

- Type of data? e.g. clinical data, OMIC or imaging data or biological samples?
- If the researchers are Academics, if they are from the pharmaceutical industry for profit, if they are researchers within the country or if they are from another country similar to ours, or if they are researchers in a high-income country?
- If the participants are a vulnerable population? Minors or adults? Aboriginal (indigenous) populations

5. What are the most important benefits for participants and their communities, derived from sharing data in an investigation?

---

[1]

Probe:

Do you think the benefits are different according to:

- type of data? e.g. clinical data, OMIC or image data or samples?
- Type of data? e.g. clinical data, OMIC or imaging data or biological samples?
- If the researchers are Academics, if they are from the pharmaceutical industry for profit? If they are researchers within the country or if they are from another country similar to ours, or if they are researchers from a high-income or developed country?
- If the participants are a vulnerable population? Minors or adults? Aboriginal populations (indigenous)

6. When you review ethics requests for studies that include broad consent for future use or exchange of data / biological samples, what elements of the study do you consider essential to be examined and to make a decision?

Probe:

- the type of study
- the population participating in the study
- the ability to anonymize data
- the type of funder, the type of researcher
- the trust you have in the funder or the researcher / research group, due to previous experience or fame
- That it has an appropriately structured and functional governance for the storage and handling of data and samples
- If there is community participation in this governance
- distribution of benefits / dissemination of results (with the study team, with the community)
- The value of research and its importance for public health.
- Whether or not the study takes place in the context of an epidemic
- Others, specify

6.b. Within these elements and those that you mention:

a. Main reasons for approval:

b. Top reasons for Disapproval:

7. What are the most important resources you use when evaluating proposals that include broad informed consent for future use?

Probe:

- Peer consultation - debate within the committee
- Community consultation
- Personal criteria

• Previous training
• Local guidance / regulations
• National guidelines / regulations
• International guidelines / regulations - CIOMS

8. What are your recommendations for researchers who wish to include broad informed consent for future use in their research protocols and informed consent forms?

9. Can waivers of consent be granted for the exchange of data or samples when the original study did not initially include broad informed consent?

a. Yes _
b. No _

10. What elements of a secondary study do you consider essential to be examined and to make a decision, in those studies that include waivers of consent for the use or exchange of data or samples, when the original study did not include broad informed consent?

Probe

Importance of:

- the type of study
- the population participating in the study
- the ability to anonymize data
- the type of funder, the type of researcher
- the confidence you have in the funder or the researcher / research group, from previous experience etc.
- that has an appropriately structured and functional governance for the storage and handling, administration of data and samples
- If there is community participation in this governance
- distribution of benefits / dissemination of results (with the study team, with the community)
- the value of research and its importance to public health.
- Whether or not the study is during an epidemic and related to it or not.
  Others, specify

11. Within these elements and those that you mention:

a. Main reasons for approval:

b. Top Reasons for Disapproval:

12. Who do you think is the “owner” or “who owns it? data and samples collected in the context of human research?

13. Researchers are often pressured by sponsors and scientific journals to share data or samples of the studies they conduct. What would you say to sponsors and scientific journals that require researchers to share unidentified data or samples at the participant level?

Guía de entrevista semiestructurada

Formación / experiencia previa

1. ¿Cuáles fueron sus motivaciones para participar en este comité de ética?

Enfoque para evaluar el consentimiento informado amplio para uso futuro de datos y muestras biológicas

1. ¿Desde su punto de vista, quiénes considera que son las partes (o actores) interesadas cuando está evaluando los protocolos de investigación que incluyen una declaración sobre el intercambio de datos no identificados para fines distintos a los descritos en el estudio de investigación original, en otras palabras, un consentimiento informado amplio para el uso futuro de los datos o muestras?

Ejemplo: Si considera que un grupo 1 de actores interesados sean: los financiadores y los investigadores y el grupo 2: los participantes en la investigación y las comunidades de donde provienen.

Indagar

- ¿En qué se diferencian los intereses de GRUPO 1 y DEL GRUPO 2?

3. Describa un momento en el que los intereses de los distintos actores involucrados en un proyecto de investigación hayan estado en conflicto.

Indagar

- ¿Cómo evaluó/o sopesó los intereses de las diferentes partes/grupos?
- ¿Qué lo ayudó a tomar una posición o una decisión frente a esos intereses?

4. ¿En su opinión cuáles son los mayores riesgos para los participantes y para sus comunidades, derivados o asociados con el hecho de que los investigadores compartan/ usen sus datos anonimizados, en otras investigaciones diferentes a la investigación original?. Que pasa cuando ese balance de riesgos es valorado diferente individualmente en el seno del comité? Teniendo en cuenta que todos somos individuos, pero las definiciones se toman en comité, Ud, sintío alguna vez que su opinión era diferente a la de la mayoría?

Indagar

- ¿Tipos de datos? p.ej. datos clínicos, OMIC o datos de imágenes o muestras biológicas?
- ¿Si los investigadores son Académicos, si son de la industria farmacéutica y/o con fines de lucro?, si son investigadores dentro del país o si son de otro país similar al nuestro, ¿o si son investigadores en un país desarrollado?
- ¿Si los participantes son población vulnerable? ¿Menores de edad o adultos? Poblaciones aborígenes (indígenas)

5. ¿Cuáles son los beneficios más importantes para los participantes y sus comunidades, asociados con el intercambio de datos en una investigación?

Indagar

- tipos de datos? p.ej. datos clínicos, OMIC o datos de imágenes o muestras?
- ¿Tipos de datos? p.ej. datos clínicos, OMIC o datos de imágenes o muestras biológicas?
- ¿Si los investigadores son Académicos, ¿si son de la industria farmacéutica con fines de lucro?, ¿si son investigadores dentro del país o si son de otro país similar al nuestro, o si son investigadores de un país de altos ingresos o desarrollado?
- ¿Si los participantes son población vulnerable? ¿Menores de edad o adultos? Poblaciones aborígenes (indígena)

6. a. Cuando revisa las solicitudes de ética de los estudios que incluyen un consentimiento informado amplio para uso futuro o intercambio de datos/muestras biológicas ¿qué aspectos del estudio considera indispensables para ser examinados y tomar una decisión?

Indagar:

• el tipo de estudio
• la población participante en el estudio
• la capacidad para anonimizar los datos
• el tipo de financiador, el tipo de investigador
• la confianza que usted tiene en el financiador o en el investigador /grupo de investigación, por experiencia previa o fama
• Que tenga una gobernanza apropiadamente estructurada y funcional para el almacenamiento y manejo de datos y muestras
•Si hay participación de la comunidad en esta gobernanza
•distribución de beneficios/diseminación de resultados (con el equipo del estudio, con comunidad)
• El valor de la investigación y la importancia para la salud pública.
• Si el estudio se desarrolla o no en el contexto de una epidemia
•Otros, especifique

6 b. Dentro los aspectos que Ud. ha mencionado:

a. Principales razones para la aprobación:

b. Principales razones para la desaprobación:

7. ¿Cuáles son los recursos más importantes que utiliza cuando evalúa propuestas que incluyen un consentimiento informado amplio para uso futuro?

Indagar

• Consulta de pares – debate dentro del comité
• Consulta comunitaria
• Criterio personal
• Formación previa
• Orientación / regulaciones locales
• Orientaciones / regulaciones nacionales
• Orientaciones / regulaciones internacionales - CIOMS

8. ¿Cuáles son sus recomendaciones para los investigadores que deseen incluir un consentimiento informado amplio para uso futuro en sus protocolos de investigación y formularios de consentimiento informado?

9. a ¿Qué aspectos de un estudio secundario considera indispensables para ser examinados y tomar una decisión, en aquellos estudios que incluyen exenciones de consentimiento para el uso o intercambio de datos o muestras, cuando el estudio original no incluía un consentimiento informado amplio?

Indagar:

a. Importancia de:

- el tipo de estudio
- la población participante en el estudio
- la capacidad para anonimizar los datos
- el tipo de financiador, el tipo de investigador
- la confianza que usted tiene en el financiador o en el investigador /grupo de investigación, por experiencia previa etc
- que tenga una gobernanza apropiadamente estructurada y funcional para el almacenamiento y manejo, administración de datos y muestras
- Si hay participación de la comunidad en esta gobernanza
- distribución de beneficios/diseminación de resultados (con el equipo del estudio, con comunidad)
- el valor de la investigación y la importancia para la salud pública.
- Si el estudio es o no durante una epidemia y relacionado o no con esta.

Otros, especifique

9. Dentro de los aspectos que Ud. ha mencionado:

- Principales Razones para la aprobación:

- Principales Razones para la desaprobación:

10. ¿Quién cree que es el “propietario” o “a quién le pertenecen los datos y las muestras colectados en el contexto de la investigación en seres humanos?

11. Los investigadores a menudo se ven presionados por los patrocinadores y las revistas científicas para compartir datos o muestras de los estudios que realizan. ¿Qué les diría a los patrocinadores y las revistas científicas que requieren que los investigadores compartan datos o muestras no identificadas a nivel de participante?
