# Supplementary material for: A blank check or a global public good? A qualitative study of how ethics review committee members in Colombia weigh the risks and benefits of broad consent for data and sample sharing during a pandemic
Source: PLOS Glob Public Health. 2022 Jun 6;2(6):e0000364. doi: 10.1371/journal.pgph.0000364 (PMC10022129; doi:10.1371/journal.pgph.0000364)
Supplement: Attachment — Submitted filename: Response to reviewers.pdf [file pgph.0000364.s004.pdf]

Lauren Maxwell, PhD, MPH
Senior Scientist

Heidelberger Institut für Global Health
Im Neuenheimer Feld 130.3
69120 Heidelberg
Fon +49 6221 56-36637
Fax +49 6221 56-5948
lauren.maxwell@uni-heidelberg.de
www.klinikum.uni-heidelberg.de

HEIDELBERG
UNIVERSITY
HOSPITAL

Heidelberg University Hospital ç Im Neuenheimer Feld 130.3 ç 69120 Heidelberg

M. Dylan Bould
Academic Editor
PLOS Global Public Health
1265 Battery Street, Suite 200
San Francisco, CA 94111
United States

18 March 2022

Dear Dr. Bould,

Thank you for presenting us with three detailed reviews of our manuscript, "A blank check or a global public good? A qualitative study of how ethics review committee members in Colombia weigh the risks and benefits of broad consent for data and sample sharing during a pandemic" and for inviting the revision and resubmission of our work. We discuss below our responses to the reviewers who we believe have further improved the manuscript.

The revised manuscript with the additional explanations requested by the reviewers contains 8882 words. We believe that these substantive additions were necessary to address the reviewers' thoughtful questions and suggested edits. Thank you for your continued interest in our paper.

RESPONSE TO THE EDITOR

I'm glad that you have referenced the Standards for Reporting Qualitative Research - but there are a few points in these guidlelines that you could pay closer attention to. In particular it is not clear if there was any particular qualitative approach or guiding theory for this work. There could be more detail in the description of the qualitative analysis and could you reference a particular paradigm or process? I'm interested in whether you used any techniques to enhance the trustworthiness and credibility of the analysis, including member checking.

Response: We appreciate the editor's excellent questions here. We have substantially revised our description of the analytic process to provide the missing detail warranted here. Bolded text was added to the analysis section to better describe the qualitative approach and theoretical underpinnings.

> The written, de-identified transcripts were uploaded to MAXQDA [1] software and analyzed using a combined deductive and inductive approach **rooted in Colaizzi's approach to descriptive phenomenology [2, 3]. During the first phase of the analysis, we developed an initial set of deductive codes and definitions based on (1) the interview guide; (2) literature identified through a concurrent scoping review related to broad consent for future use [4] and (3) our own experience in research ethics as members of ERCs and as researchers in the field of public health ethics and data sharing.** Inductive codes were identified through a review of the written transcripts and interviewer field notes. **Transcripts were read several times so that the team could familiarize themselves with the data and identify important**

UK
HD

Lauren Maxwell, PhD, MPH
Senior Scientist

Heidelberger Institut für Global Health
Im Neuenheimer Feld 130.3
69120 Heidelberg
Fon +49 6221 56-36637
Fax +49 6221 56-5948
lauren.maxwell@uni-heidelberg.de
www.klinikum.uni-heidelberg.de

statements which were then interpreted by the research team. All authors reviewed three transcripts together to refine the codebook and definitions. To ensure consistency in the interpretation of codes, two researchers (JBC, LML) coded several additional interview transcripts together. To ensure reliability, codes were independently applied to blocks of text in the interview transcripts by two researchers (JBC, LML) who met weekly to discuss and resolve differences in the application of codes and discuss emergent inductive codes, grounded in the transcript data. During the same period, the entire team held weekly discussions to update and refine the codebook and to ensure agreement on the application of the codes across all team members. This process meant that two team members independently reviewed all transcripts and a subset of transcripts was reviewed and evaluated by all four members of the team. Team members documented the evolution of codes through memos and meeting notes and compared the application of codes in later transcripts to that of earlier transcripts to ensure consistent application of codes (reliability) throughout the first phase of the analysis. Earlier interviews were recoded to account for emerging codes and evolving definitions.

During the second phase of the analysis, the team met weekly to group the codes into meaningful themes and to discuss whether codes and related themes varied across participants of different genders, levels of experience on ERCs, and positions within the ERC. Reliability was ensured through weekly meetings to discuss the evolution and application of codes and their definitions between the coders and with the entire team. The interview guide was modified to explore emerging themes, **including the tension between an individual's beliefs and values and the group's beliefs and values. Validity was ensured through grounding deductive codes in the findings from a concurrent scoping review and team members' own experience as ERC members and researchers in the field of bioethics and data and sample sharing and in the use of the interview transcripts to identify emerging (inductive) codes. Throughout the project, differences of opinion in the development and application of codes and themes were resolved by consensus.** Results are presented in keeping with the Standards for Reporting Qualitative Research [5]. The IDI guide is included as S1 File.

We did not plan to and did not engage in member checking for the following reasons:

1. we engaged in other methods to validate our findings

We included intercoder reliability exercises to ensure consistency in the interpretation of codes. First, all authors reviewed several transcripts together to refine the codebook and definitions. Second, two researchers (JBC, LML) coded several additional interview transcripts together to ensure consistency in the interpretation of the codebook and style of coding. Remaining transcripts were independently coded and once each transcript had been completed, coders met and compared their codes and coding decisions to determine whether there were any inconsistencies, which were typically resolved by negotiated consensus. Finally, all co-authors met weekly during the analysis to review and update definitions and interpretation of existing codes and discuss emergent themes.

HEIDELBERG UNIVERSITY HOSPITAL

Lauren Maxwell, PhD, MPH
Senior Scientist

Heidelberger Institut für Global Health
Im Neuenheimer Feld 130.3
69120 Heidelberg
Fon +49 6221 56-36637
Fax +49 6221 56-5948
lauren.maxwell@uni-heidelberg.de
www.klinikum.uni-heidelberg.de

Heidelberg University Hospital ç Im Neuenheimer Feld 130.3 ç 69120 Heidelberg

2. we were concerned about social desirability bias

ERC members, like the rest of the public health research ecosystem, are under pressure from funders and researchers to enable data and sample sharing. Given these external pressures, to best understand ERC members' concerns, we took a number of steps to prevent social desirability bias. These included: (1) assuring participants that any identifying information would be removed from written transcripts; (2) telling participants that there were no "right" answers; (3) using open ended questions; (4) revising the interview guide to remove language that would indicate bias in favor of (or against) data and sample sharing. Despite these prospective efforts, we found that ERC members indicated that they felt they would be "judged" for their responses both at the recruitment stage, where we were asked to make a formal presentation to the ERC of the study protocol and ERCs took a collective decision on whether to participate or not and recommended certain ERC members to participate and as part of the interview process, where participants indicated through their tone or through comments before or after the interview that they felt judged by some of the questions, for example, "how do you define broad consent for future use."

We believe that there are several reasons for this: (1) as stated in the protocol, our team decided we needed to present the protocol formally to ERCs which may have lent itself to the perception that the research itself was used to evaluate individuals' knowledge or attitudes towards broad consent for future use; (2) our research team included a representative from the Colombian Ministry of Science, Technology and Innovation, which we realized caused some participants to feel that they needed to "study" regulations for broad consent for future use as the evaluation of ERCs is part of the mandate of the Ministry of Science, Technology and Innovation; (3) globally, there has been a push by WHO and major funders (BMBF, NIH/NIAID, Wellcome, EU, etc.) to mandate data (and sometimes sample) sharing.

3. our research team included members of the research subject source community

Our research team was composed of members of the same community as the participants themselves. Both interviewers and the first author have served on ERCs or regularly interact with ERCs within Colombia. Given that the study team comes from the same population (education level, training, etc.), we felt that it was unlikely we would misinterpret participants' statements related to how they approach broad consent for future use.

4. We used participants' own language to present findings

Our presentation of the results privileges participants' actual language. We took care to include both the original Spanish language quotes and the English-language translations to allow readers fluent in both to evaluate the translation.

Lauren Maxwell, PhD, MPH
Senior Scientist

Heidelberger Institut für Global Health
Im Neuenheimer Feld 130.3
69120 Heidelberg
Fon +49 6221 56-36637
Fax +49 6221 56-5948
lauren.maxwell@uni-heidelberg.de
www.klinikum.uni-heidelberg.de

In summary, we think that the pressure to report the "right" answers, the connections between participants' responses and the emergent themes in the written analysis and supplement, and the independent coding of transcripts by two reviewers, supplemented by group discussions of the analysis support our decision to not engage in member checking.

JOURNAL REQUIREMENTS

We have included the PLOS questionnaire on inclusivity in global research in our revised manuscript and addressed all other Journal requirements.

RESPONSE TO REVIEWER 1

This is a good article that addresses a topic of the utmost importance that needs to be urgently regulated by the States. In this sense, the research offers an inventory of aspects that need to be taken into account in the elaboration of this regulation. It is also very important that qualitative studies are valued and that articles on issues related to research ethics are encouraged. Another positive aspect of the text is to give voice to members of ethics committees, which is very rare in the literature. The question elaborated in the title corresponds to a good research problem. The qualitative research seems well planned and executed.

However, some doubts remain. The first one would be: why Colombia? Since Plos Global Public Health is an international publication, it would be important to explain to readers whether Colombia is a country that can be considered a standard or an exception regarding the legal treatment of this issue, but also regarding the work of the ERC and scientific research. Do most States have rules or not? Is the fact that Colombia is a developing country related to the lack of regulation of the matter pointed out by the authors? In other words, contextualizing the situation of the chosen country in the global scenario would contribute to a better use of the results.

Response: We would like to thank Reviewer 1 for their feedback on our manuscript. Other than the fact that our research team includes several Colombian researchers and philosophers who focus on research ethics or participate in ERCs, we have added several paragraphs to the introduction to answer the question: Why Colombia? With these additions, we have tried to better describe how this research study, conducted in Colombia, has implications for other countries in Latin America and the Caribbean (LAC) and elsewhere. Text in bold has been added to address this feedback.

The legal and policy context for broad consent in Latin America and the Caribbean

A 2018 Pan American Health Organization (PAHO)-led meeting of Latin American and the Caribbean (LAC) countries identified developing a systematic approach to research ethics, that included capacity building and guidelines for ERC review during public health

HEIDELBERG
UNIVERSITY
HOSPITAL

Lauren Maxwell, PhD, MPH
Senior Scientist

Heidelberger Institut für Global Health
Im Neuenheimer Feld 130.3
69120 Heidelberg
Fon +49 6221 56-36637
Fax +49 6221 56-5948
lauren.maxwell@uni-heidelberg.de
www.klinikum.uni-heidelberg.de

Heidelberg University Hospital ç Im Neuenheimer Feld 130.3 ç 69120 Heidelberg

emergencies, as a regional priority, but neither broad consent nor data or sample sharing were mentioned in the related meeting resolution [6]. A recent review of national guidance from 19 of the 33 countries in LAC related to polices for expediting review of COVID-19-related human subjects research during the pandemic found that Colombia was among the 10 LAC countries that implemented such guidance [7]. While future use was among the topics considered in six LAC countries that issued COVID-19-related guidance to ERCs, Colombia was not one of these countries [7]. These findings reflect a similar policy analysis conducted in 22 African countries which found that only three countries had national or local guidelines related to biobanking and genetic research [8]. Three African countries had laws or guidelines related to broad consent for future use, which was not allowed or required reconsenting subjects in those countries [8].

The legal context for broad consent in Colombia

Colombia has a national legal framework for evaluating ethical concerns related to the interface of research and medical practice but does not have a national legal framework to guide the consideration of protocols that include broad consent for future use. **That said, the Colombian legislature has been working to regulate the establishment of biobanks and the use of samples and biological collections in research [9].** The bill "By means of which the operation of Biobanks for biomedical research purposes is regulated and other provisions are enacted" ("Por medio del cual se regula el funcionamiento de los Biobancos con fines de investigación biomédica y se dictan otras disposiciones"), which addresses the ownership and sharing of samples, was filed by the legislature in 2017 [9] but has not been considered further. **Subsequently, legislative proposal 168, to"regulate the constitution and operation of biobanks for obtaining, processing, storage, transport and transfer of human biological samples, their derivatives, associated clinical and biological information for biomedical research purposes" was submitted for review by the Senate of Colombia in 2019.**

Regarding the opinion of ERC members, at the end of the text the authors write: "Similar to the findings from a stakeholder meeting with ERC members in Africa, ERC members in Colombia were supportive of the concept of broad consent for future use of data and samples given transparent governance that fairly weights community and research participant concerns and benefits [20]"(603-606) and "Concerns expressed by ERC members in Colombia reflect those of research participants in diverse contexts and from ERC members in Africa and the US" (618-619). This point which would be one of the major contributions of the paper, but was the subject of only two sentences that are based on only one reference. The idea is floated but not developed.

Response: We thank the reviewer for their insightful comments here. We agree that we should have better described these points. We added the following text to the discussion section to address the reviewer's comment.

ERCs' chief role is to protect the rights of research participants and ensure the social value of research through evaluating research quality and weighing the potential benefits and harms associated with research. Multicenter trials and cross-national research present

HEIDELBERG UNIVERSITY HOSPITAL

Lauren Maxwell, PhD, MPH
Senior Scientist

Heidelberger Institut für Global Health
Im Neuenheimer Feld 130.3
69120 Heidelberg
Fon +49 6221 56-36637
Fax +49 6221 56-5948
lauren.maxwell@uni-heidelberg.de
www.klinikum.uni-heidelberg.de

Heidelberg University Hospital ç Im Neuenheimer Feld 130.3 ç 69120 Heidelberg

ethical, legal, and logistic challenges for investigators and ERCs in LMIC countries [8, 10]. Many LMICs, including Colombia, do not have national legislation or clear policies related to broad consent for future use for data and samples [8], including sharing data or samples with other countries. The key ethical principles of justice, autonomy, beneficence, and nonmaleficence may be interpreted differently in different contexts [11]. For example, the intervention [12] and protection [13] models of bioethics, which privilege the role of shared (public) health concerns over individual rights and consider health justice in the presence of scarcity with a focus on vulnerable populations [14], emerged from LAC [15] and can be contrasted with principalism, which emerged from Europe and is rooted in individual rights [16].

Primary research on how ERCs evaluate human subjects research applications that include broad consent for future use is extremely limited. We searched Ovid(Medline) from 1 January 2000 to 21 November 2021 using the following title and abstract text terms: ((future use* OR (broad OR blanket OR wide OR open OR data shar*) adj2 (consent OR data shar*)) AND ((ethics adj2 committee* OR 3 institutional review board* OR ethics committee* OR research ethics OR ethics review)) to identify existing primary research related to ERC members consideration of broad consent for future use. We only identified two qualitative research studies that explored broad consent-related knowledge, attitudes, or practices of ERC members in Malawi (N=11) [17] and the US (N=3) [18] and a meeting report from ERCs in 15 African countries (N=41) [19]. Concerns expressed by ERC members in Colombia in this study reflect the findings of these two studies and the meeting report. While the similarities across these three small studies cannot be the basis for any type of generalization, they do suggest a convergence of ERC members' attitudes towards broad consent for future use that should be further explored and addressed. As the first study to address how Colombian ERCs weigh different stakeholders' concerns when reviewing protocols that include broad informed consent, and one of only two other research studies on the topic in any country, provides insight into the challenges faced by ERC members when evaluating provisions in the IC that facilitate future collaborations between countries, including between developed and developing countries.

In the absence of national regulation, LMIC may default to laws and policies enacted in different countries or regions, including the GDPR, data sharing legislation enacted in Europe, and the CIOMS guidance [20], which includes considerations for broad consent for future use. Understanding how ERCs make decisions about future use in the absence of national legislation is important for understanding how ERC members weigh the considerations of different stakeholders when making decisions about broad consent for future use and has been flagged as a concern by other groups [21]. Even when there are national laws or policies, some studies indicate that ERC members' decision making may not correspond to those [22, 23]. While the presence of national legislation doesn't necessarily mean that ERC members' evaluations of broad consent for future use in similar protocols for similar purposes will be aligned, encoding national values and preferences would likely help with such alignment. Given ERC members' importance as research gatekeepers, future

Lauren Maxwell, PhD, MPH
Senior Scientist

Heidelberger Institut für Global Health
Im Neuenheimer Feld 130.3
69120 Heidelberg
Fon +49 6221 56-36637
Fax +49 6221 56-5948
lauren.maxwell@uni-heidelberg.de
www.klinikum.uni-heidelberg.de

> **research is needed to understand ERC decision making processes around broad consent for future use regardless of the presence of national legislation.**

Also missing is the reference to other articles that address exactly the main issue of the research during an international emergency - for example Alirol, E., Kuesel, A. C., Guraiib, M. M., de la Fuente-Núñez, V., Saxena, A., & Gomes, M. F. (2017). Ethics review of studies during public health emergencies - the experience of the WHO ethics review committee during the Ebola virus disease epidemic. BMC medical7 ethics, 18(1), 43. https://doi.org/10.1186/s12910-017-0201-1.

Response: We agree with the author that we should have cited ongoing work in this area. We have added the following text to the discussion section:

> **At the same time, international ethics bodies have called for the prospective consideration of broad consent for future use of data and samples during public health emergencies [24, 25].**

In summary, I believe it is essential that the article devotes a few paragraphs to the global context, both with regard to national standards and with regard to the views of ERC members from other countries, as this literature already exists and can be consulted.

Response: We agree with this comment and hope the changes mentioned above sufficiently address this important point.

Finally, I believe that the question formulated in the title of the article is not sufficiently answered in the final considerations which could be improved.

Response: We have added an additional paragraph to the conclusion to better address this point.

> **ERC members described the continuum along which they evaluate broad consent for future use where data and sample sharing can be both a Trojan horse and a global public good. The steps that researchers take to specify the governance structure and organizations who will manage future data and/or sample sharing, how researchers engage the community to determine preferences for future use, how well ERC members think participants will understand the subject information sheet and informed consent, future use by commercial entities, etc. all help determine how broad consent is perceived by ERC members. Several frameworks for ethics review in public health emergencies have highlighted the need for ERCs to address data and sample sharing [24]. ERC members' concerns reflect a subsequent call from the WHO to align and support equitable governance structures to realize health research data as a global public good [26]. As such, study findings have implications for researchers and ERCs in Colombia and in other countries that do not have a legislative framework for broad consent for future use or data and sample sharing during the ongoing COVID-19 pandemic and beyond.**

HEIDELBERG UNIVERSITY HOSPITAL

Lauren Maxwell, PhD, MPH
Senior Scientist

Heidelberger Institut für Global Health
Im Neuenheimer Feld 130.3
69120 Heidelberg
Fon +49 6221 56-36637
Fax +49 6221 56-5948
lauren.maxwell@uni-heidelberg.de
www.klinikum.uni-heidelberg.de

Heidelberg University Hospital ç Im Neuenheimer Feld 130.3 ç 69120 Heidelberg

RESPONSE TO REVIEWER 2

In this cross-sectional qualitative study using a semi-structured interview of a broad range of members of Ethical Review Boards in Colombia, authors circumscribed the research question of the perception of the ERCs' members on the broad consent for future use. Perceived risks and potential benefits at research participant-level, community level and to more extent benefits and risks when sharing data or samples are operated between LMICs and high income countries including data used for profit purposes are explored. The interviews did not omit to include the perception of this issue with regard to the current COVID-19 pandemic. This study is of great interest as it raises concerns surrounding this topic and draws some potential orientation for solutions including the governance and need to broaden information on possible use of future researches of data or samples in order to obtain an authentic informed consent for participants and guarantee of data safeguarding.

I hereby identified some minor comments: - Line 100: …related to instead of…..relate to… - Line 120: ….interventional instead of …. intervention –

Table 1: use Male and Female …..instead of….. Masculine and Feminine

Occupation may rather be replaced by Professional background –

Response: We thank the reviewer for their close read of the text. We have implemented the aforementioned changes.

Line 202: ..selected protocols instead of select protocols

Response: This was changed to "some protocols" to retain the meaning.

Lines 114-116: The research team reviewed related literature and consulted with researchers who work on data and sample sharing in a number of different LMICs and globally. The role or impact of this review on this study should be well mentioned in Methods.

Response: We added the following text to the methods section to address this oversite:

> **The external review resulted in (1) the rewording of the study objective in the participant information sheet to take a more neutral approach towards broad consent for future use and data and sample sharing more generally and (2) the reorganization of the interview guide, including the removal of redundant questions and a reduction in the number of probes.**

Lines 138-150: around the data collection (interviewers) especially the philosopher… If these researchers are among the authors for this manuscript, the initials of their names should be mentioned in parentheses to ease the riders' work. Thank you for good and commendable piece of work

HEIDELBERG UNIVERSITY HOSPITAL

Lauren Maxwell, PhD, MPH
Senior Scientist

Heidelberger Institut für Global Health
Im Neuenheimer Feld 130.3
69120 Heidelberg
Fon +49 6221 56-36637
Fax +49 6221 56-5948
lauren.maxwell@uni-heidelberg.de
www.klinikum.uni-heidelberg.de

Heidelberg University Hospital ç Im Neuenheimer Feld 130.3 ç 69120 Heidelberg

Response: Team members' initials were added to the text in the Methods section.

RESPONSE TO REVIEWER 3

Excellent paper. It addresses an important emerging area of policy and practice and the analysis is well supported by the research. This will surely make a substantive contribution to inform and further the development of research ethics standards and guidelines for broad consent for future uses of data.

Response: We thank the reviewer for their commendation.

We hope that our responses have adequately addressed the reviewers' concerns. We have uploaded the revised version of the manuscript and other related documents as directed.

If you have further questions, please do not hesitate to contact us. Thank you in advance for your consideration. We look forward to hearing from you.

With warm regards,

Lauren Maxwell
on behalf of all authors

References

1. Plus M. MAXQDA. Berlin, Germany: VERBI Software GmbH; 2020.
2. Morrow R, Rodriguez A, King NJTp. Colaizzi's descriptive phenomenological method. 2015;28(8):643-4.
3. Colaizzi PF. Psychological research as the phenomenologist views it. In: Valle RS, King M, editors. Existential-Phenomenological Alternatives for Psychology: Oxford University Press; 1978.
4. Maxwell L, Gilyan R, Chavan SA, Merson L, Saxena A, Terry R. Guidance for ensuring fair and ethical broad consent for future use. A scoping review protocol. F1000Research. 2021;10:102-. doi: 10.12688/f1000research.51312.1. PubMed PMID: 33953907.

5. O’Brien BC, Harris IB, Beckman TJ, Reed DA, Cook DA. Standards for Reporting Qualitative Research: A Synthesis of Recommendations. Academic Medicine. 2014;89(9):1245-51. doi: 10.1097/acm.0000000000000388. PubMed PMID: 00001888-201409000-00021.
6. Bioethics: towards the integration of ethics in health. Resolution CSP28.R1. Washington, D.C.: Pan American Health Organization, 2018 September 23–27, 2018. Report No.
7. Palmero A, Carracedo S, Cabrera N, Bianchini A. Governance frameworks for COVID-19 research ethics review and oversight in Latin America: an exploratory study. BMC Medical Ethics. 2021;22(1):147. doi: 10.1186/s12910-021-00715-2.
8. de Vries J, Munung SN, Matimba A, McCurdy S, Ouwe Missi Oukem-Boyer O, Staunton C, et al. Regulation of genomic and biobanking research in Africa: a content analysis of ethics guidelines, policies and procedures from 22 African countries. BMC Medical Ethics. 2017;18(1):8. doi: 10.1186/s12910-016-0165-6.
9. Gunturiz Albarracín M, Castro-Osorio C. Biobancos y COVID-19: implicaciones éticas. Revista Colombiana de Bioética. 2020;15(1). doi: https://doi.org/10.18270/rcb.v15i1.3065.
10. Ervin A-M, Taylor HA, Meinert CL, Ehrhardt S. Evidence gaps and ethical review of multicenter studies. Science. 2015;350(6261):632-3. doi: DOI: 10.1126/science.aac4872.
11. Haidt J, Graham JJSJR. When morality opposes justice: Conservatives have moral intuitions that liberals may not recognize. 2007;20(1):98-116.
12. Porto D, Garrafa VJRB. Bioética de intervenção: considerações sobre a economia de mercado. 2005;13(1):111-23.
13. Schramm FRJRB. Bioética da proteção: ferramenta válida para enfrentar problemas morais na era da globalização. 2008;16(1):11-23.
14. Schramm FRJC, Coletiva S. Bioethics of protection: a health practice evaluation tool? 2017;22(5):1531-8. doi: 10.1590/1413-81232017225.04532017.
15. Garcia LF, Fernandes MS, Moreno JD, Goldim JR. Mapping Bioethics in Latin America: History, Theoretical Models, and Scientific Output. J Bioeth Inq. 2019;16(3):323-31. Epub 2019/02/20. doi: 10.1007/s11673-019-09903-7. PubMed PMID: 30778904.
16. Callahan D. Principlism and communitarianism. 2003;29(5):287-91. doi: 10.1136/jme.29.5.287 %J Journal of Medical Ethics.
17. Matandika L, Ngóngóla RT, Mita K, Manda-Taylor L, Gooding K, Mwale D, et al. A qualitative study exploring stakeholder perspectives on the use of biological samples for future unspecified research in Malawi. BMC Medical Ethics. 2020;21(1):61. doi: 10.1186/s12910-020-00503-4.
18. Mazor K, Richards A, Gallagher M, Arterburn DE, Raebel MA, Nowell WB, et al. Stakeholders’ views on data sharing in multicenter studies. 2017;6(6):537-47. doi: 10.2217/cer-2017-0009. PubMed PMID: 28805448.
19. de Vries J, Littler K, Matimba A, McCurdy S, Ouwe Missi Oukem-Boyer O, Seeley J, et al. Evolving perspectives on broad consent for genomics research and biobanking in Africa. Report of the Second H3Africa Ethics Consultation Meeting, 11 May 2015. Global Health, Epidemiology and Genomics. 2016;1:e13. Epub 2016/07/25. doi: 10.1017/gheg.2016.5.
20. (CIOMS) CfIOoMS. International ethical guidelines for health-related research involving humans. Geneva, Switzerland: 2016 9290360887.
21. Seykora A, Coleman C, Rosenfeld SJ, Bierer BE, Lynch HF. Steps toward a System of IRB Precedent: Piloting Approaches to Summarizing IRB Decisions for Future Use. 2021;43(6):2-18. doi: https://doi.org/10.1002/eahr.500106.
22. Klitzman R. The Ethics Police?: IRBs' Views Concerning Their Power. PLOS ONE. 2011;6(12):e28773. doi: 10.1371/journal.pone.0028773.

23. Silberman G, Kahn KL. Burdens on Research Imposed by Institutional Review Boards: The State of the Evidence and Its Implications for Regulatory Reform. 2011;89(4):599-627. doi: https://doi.org/10.1111/j.1468-0009.2011.00644.x.
24. Alirol E, Kuesel AC, Guraiib MM, de la Fuente-Núñez V, Saxena A, Gomes MF. Ethics review of studies during public health emergencies - the experience of the WHO ethics review committee during the Ebola virus disease epidemic. BMC Medical Ethics. 2017;18(1):43. doi: 10.1186/s12910-017-0201-1.
25. Ethical standards for research during public health emergencies: distilling existing guidance to support COVID-19 R&D. Geneva, Switzerland: World Health Organization, 2020.
26. Health Data as a global public good – a call for Health Data Governance 30 September Geneva, Switzerland: World Health Organization; 2021 [updated September 29, 2021; cited 2022 March 14]. Available from: https://www.who.int/news-room/articles-detail/health-data-as-a-global-public-good-a-call-for-health-data-governance-30-september.
